# Social Cognitive Determinants of Nutrition and Physical Activity Among Web-Health Users Enrolling in an Online Intervention: The Influence of Social Support, Self-Efficacy, Outcome Expectations, and Self-Regulation

**DOI:** 10.2196/jmir.1551

**Published:** 2011-03-17

**Authors:** Eileen Smith Anderson-Bill, Richard A Winett, Janet R Wojcik

**Affiliations:** ^2^Exercise Science ProgramDepartment of Physical Education, Sports & Human PerformanceWinthrop UniveristyRock Hill, SCUnited States; ^1^Center for Research in Health BehaviorDepartment of PsychologyVirginia TechBlacksburg, VAUnited States

**Keywords:** Internet users, dietary habits, physical activity, psychosocial aspects, self-efficacy, social support, self-regulation

## Abstract

**Background:**

The Internet is a trusted source of health information for growing majorities of Web users. The promise of online health interventions will be realized with the development of purely online theory-based programs for Web users that are evaluated for program effectiveness and the application of behavior change theory within the online environment. Little is known, however, about the demographic, behavioral, or psychosocial characteristics of Web-health users who represent potential participants in online health promotion research. Nor do we understand how Web users’ psychosocial characteristics relate to their health behavior—information essential to the development of effective, theory-based online behavior change interventions.

**Objective:**

This study examines the demographic, behavioral, and psychosocial characteristics of Web-health users recruited for an online social cognitive theory (SCT)-based nutrition, physical activity, and weight gain prevention intervention, the Web-based Guide to Health (WB-GTH).

**Methods:**

Directed to the WB-GTH site by advertisements through online social and professional networks and through print and online media, participants were screened, consented, and assessed with demographic, physical activity, psychosocial, and food frequency questionnaires online (taking a total of about 1.25 hours); they also kept a 7-day log of daily steps and minutes walked.

**Results:**

From 4700 visits to the site, 963 Web users consented to enroll in the study: 83% (803) were female, participants’ mean age was 44.4 years (SD 11.03 years), 91% (873) were white, and 61% (589) were college graduates; participants’ median annual household income was approximately US $85,000. Participants’ daily step counts were in the low-active range (mean 6485.78, SD 2352.54) and overall dietary levels were poor (total fat g/day, mean 77.79, SD 41.96; percent kcal from fat, mean 36.51, SD 5.92; fiber g/day, mean 17.74, SD 7.35; and fruit and vegetable servings/day, mean 4.03, SD 2.33). The Web-health users had good self-efficacy and outcome expectations for health behavior change; however, they perceived little social support for making these changes and engaged in few self-regulatory behaviors. Consistent with SCT, theoretical models provided good fit to Web-users’ data (root mean square error of the approximation [RMSEA] < .05). Perceived social support and use of self-regulatory behaviors were strong predictors of physical activity and nutrition behavior. Web users’ self-efficacy was also a good predictor of healthier levels of physical activity and dietary fat but not of fiber, fruits, and vegetables. Social support and self-efficacy indirectly predicted behavior through self-regulation, and social support had indirect effects through self-efficacy.

**Conclusions:**

Results suggest Web-health users visiting and ultimately participating in online health interventions may likely be middle-aged, well-educated, upper middle class women whose detrimental health behaviors put them at risk of obesity, heart disease, some cancers, and diabetes. The success of Internet physical activity and nutrition interventions may depend on the extent to which they lead users to develop self-efficacy for behavior change, but perhaps as important, the extent to which these interventions help them garner social-support for making changes. Success of these interventions may also depend on the extent to which they provide a platform for setting goals, planning, tracking, and providing feedback on targeted behaviors.

## Introduction

A high proportion (83% [1]) of Internet users go to the Web for information on health topics [1-3] including exercise (38% in 2008, up from 21% in 2002) and weight loss (33% in 2008). Although community, health system, and workplace health programs have effectively utilized the Internet for a wide array of behavior-change interventions, the reach of the Internet will be realized through the development of theory-based, purely online interventions for Web-health users [4,5]. Much work remains in developing sound methodology for testing the efficacy of programs delivered online [4].

Despite almost universal Internet access and adoption, researchers know little about Web-health users—the adults who go to the Web to find health behavior and behavior change information and who form the likely participant pool for online health promotion and disease prevention research. Overall, Internet users have been equally either male or female and have tended to be somewhat younger, better educated, and to have higher incomes than the general population [2,3]. Web-health users may be more likely to be female than general Internet users, and those going to the Web for health programs may have poor to fair general health [1]. To our knowledge there have been no studies examining the health behavior and related psychosocial characteristics of potential participants of entirely online health interventions.

Generally, attrition in Internet-based health programs is high at 43% to 50% [5], but these figures pertain to participants in programs that use the Internet to deliver programs as part of workplace, primary care, or other community-based interventions. Little is known about how participants interact with stand-alone Web-based health programs, that is, programs that recruit, assess, and intervene entirely online, although early studies have suggested that attrition from such studies may be higher [4]. Similarly, Internet interventions in general tend to recruit many tentative users who attempt but quickly withdraw from programs, fewer short-term users who seem to drop out after using the program for a period, and few stable users who stick with a program over the long-term [6]. With some early evidence that rates of recruitment among Web users making contact with online programs may be low (eg, 8% in a study by Murray et al [4]), it is not clear how adoption or adherence patterns apply or if these patterns are related to participants’ demographic, behavioral, or psychosocial traits.

**Figure 1:**
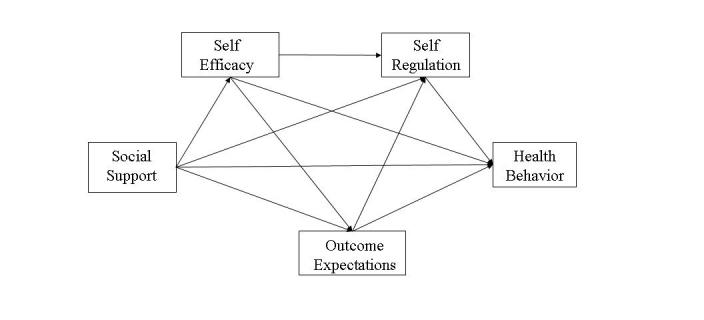
Social cognitive model of health behavior

In addition to reflecting potential participants’ characteristics, Web-based health programs should be theory-based and evaluated to validate and refine the application of theory within the Web environment [7-14]. Social cognitive theory (SCT) [15,16] is widely used as the theoretical basis for health behavior change interventions [12] suggesting Internet health interventions must help individuals develop a sense of self-efficacy in specific behaviors (such as being physically active and eating nutritiously), which stems from physically and socially supportive environments and promotes individuals’ positive expectations for behavior change. Higher levels of self-efficacy and expectations of positive outcomes lead to the modification or differential use of self-regulatory skills (ie, planning, self-monitoring, problem solving, self-standards, goals, and self-incentives) essential to maintaining behavior change (see Figure 1 for a schematic representation of SCT). Estimating the initial psychosocial characteristics of users is, therefore, essential to developing effective programs.

In previous research, self-efficacy has been associated with healthy nutrition [15,17-21] and physical activity [20,22,23] habits, as has social support from important others, such as family and friends [22,24-26]. Although outcome expectation has been found to contribute beyond self-efficacy to healthy eating habits [17-20], it has not been a consistent predictor of physical activity [27], with some studies suggesting strong support and others revealing a null effect [20,22]. Among people who desire a healthier lifestyle and who have access to healthy foods and infrastructure for physical activity, SCT suggests their success at maintaining behavior change will be determined largely by how well they set goals, plan, and monitor, that is, self-regulate such changes. Outside the obesity and weight-management literatures, self-regulation of nutrition has received scant attention and has often been poorly defined [28]. Nevertheless, self-regulatory behavior has been associated with healthier eating [10,19,22,29-33] and with promoting healthier activity levels in adults [20,22, 34].

The purpose of the present study was to examine the social cognitive determinants of nutrition and physical activity among Web-health users enrolling in a purely online SCT-based nutrition, physical activity, and weight-gain prevention intervention.

## Methods

### Recruitment and Participants

Web-health users were recruited entirely online for a clinical trial of the Web-based intervention called Guide to Health (WB-GTH) (clinical trials identifier NCT00128570). Advertisements in print and online newspapers in the major media markets of Virginia, Virginia Tech alumni publications, and online solicitations through employer and alumni-related listservs during 3 different time periods created 3 waves of recruitment: September 15, 2007 through January 23, 2008; May 8, 2008 through June 15, 2008; and July 9, 2008 through September 19, 2008. One month of Web-browser ads and 2 local direct mailings were used in wave 1 of the recruitment but yielded very few (ie, < 10) visits to the WB-GTH recruitment website. Print and online newspapers yielded some recruits, but the most effective recruitment strategy was through online alumni and employer publications and listservs. Advertisements and solicitations described the need for participants “18 to 63 years old, residing in the United States or Canada, within our weight guidelines, in good health, and not currently active” for an 18-month research project designed to test an Internet program for improving nutrition and physical activity and prevent weight gain. The Internet program was described as including a walking program “designed for you every step of the way,” a nutrition program “tailored to your needs and preferences,” and a “free pedometer and digital scale.” Preventing weight gain (not weight loss) was emphasized. Potential recruits were informed that involvement in the WB-GTH study would require them to log into the Internet program once a week for 18 months and to complete 3 two-hour assessments. Finally, recruitment materials advised potential participants that in order to be screened for study eligibility they would need to select a user id and password and provide an email address.

Approximately 4700 Internet users visited the WB-GTH site to review project information. About 15% (705) progressed no further than the GTH information page, but during the 3 recruitment waves, 3944 individuals registered for screening: 3024 during the first wave of recruitment, 364 during the second wave, and 556 during the third wave. Registering participants had a mean (SD) age of 42.54 years (12.05 years) and a mean (SD) body mass index of (BMI) of 30.81 (7.32) and were predominantly female (3311 or 84%). Based on self-report, of the 3944 individuals who registered, 88% (3454) were white, 6% (240) were African American; 4% (138) were Asian, and 3% (122) were other. In total, 3% (122/3944) reported Hispanic background. 

#### Eligible Web Users

**Figure 2:**
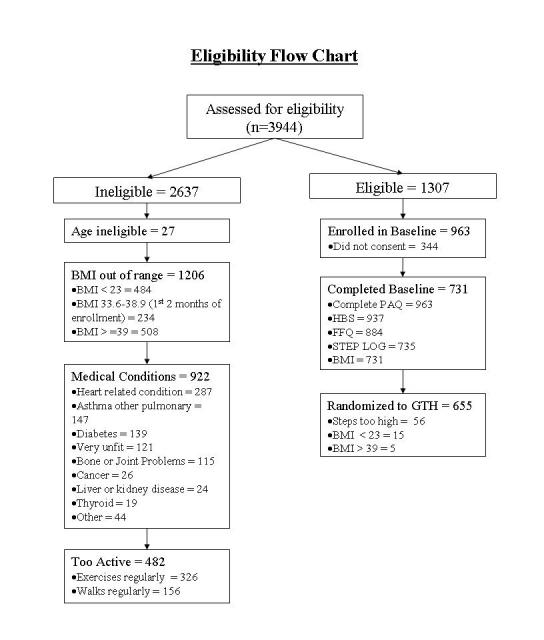
Social cognitive model of fiber, fruits and vegetables among web-health users. * *P* < .05, ** *P* < .01, *** *P* < .001

Of screened Web users, about one-third (1307) met eligibility requirements, that is, they were 18 to 63 years of age (or under 65 at the end of the trial), had high normal to obese BMI (ie, BMI 23 to 39, expanded from BMI 23 to 33 in wave 1, which was deemed unnecessarily stringent), were not currently active (ie, they did not exercise at least 20 minutes 3 times a week), but were otherwise healthy (see Figure 2). The WB-GTH program included a fitness walking component that encouraged participants to gradually move into more vigorous levels of walking exertion; hence, individuals with diagnosed coronary, metabolic or pulmonary disease, or coronary artery disease risk factors as specified by the American College of Sports Medicine [35] were excluded from the sample. Eligible participants had a mean (SD) age of 42.17 (11.17) and were predominantly female (1060 or 81%). Based on self-report, 90% (1177) of the 1307 eligible participants were white, 5% (71) were African American, 2% (21) were Asian, and 3% (38) were other. In total, 3% (34/1307) reported Hispanic background. Of the 1307 eligible Web users, 15% (203) were normal weight (BMI 23 to 24.99), 41% (532) were overweight (BMI 25 to 29.99), 33% (433) were mildly obese (BMI 30 to 34.99), and 11% (139) were obese (BMI 35 to 39.99).

#### Ineligible Web Users

Of Web users screened for the project, two-thirds (2637/3944) did not qualify. A small proportion had overlooked the age requirements listed on the information webpage and were either too old for the research project (n = 24) or declined to provide their ages (n = 3). Almost half of ineligible users did not meet the study’s weight requirements (1206/2637, 46%). The WB-GTH was designed for adults in the high normal to obese weight range so some screened participants were below the weight guidelines (BMI < 23, n = 464), but most who were ineligible were too heavy (n = 742). (As noted above, the BMI cutoff of > 32.9 was modified to BMI ≥ 39 during wave 1 recruitment). A total of 36% (1404/3944) of those who registered were excluded because of medical conditions (n = 922) or because they were too active (n = 482) (see Figure 2 for details). The mean age of ineligible Web users was 42.75 years (SD 12.45 years), similar to eligible users (F_1,3942_ = 2.40, *P* = .12), but ineligible recruits were more likely to be female (85% vs 81%, χ^2^_1_ = 11.88, *P* = .001) and of nonwhite race/ethnicity (13.6% vs 10%; χ^2^_5_ = 26.05, *P* < .001). Although ineligible users were heavier than those who were eligible with a mean (SD) BMI of 31.36 (8.49) versus a mean (SD) BMI of 29.5 (4.13) (*F*_1, 3915_ = 62.87, *P* < .001), the entire range of weights were represented in the ineligible sample, that is, 18% (477) of the 2637 ineligible Web users had a BMI less than 23, 8% (200) had a BMI from 23 to 24.99, 22% (593) had a BMI from 25 to 29.99, 20% (527) had a BMI from 30 to 34.99, 15% (387) had a BMI from 35 to 39.99, and 17% (453) had a BMI ≥ 40.

### Measures

Participants completed demographic information, physical activity, and psychosocial questionnaires on the WB-GTH website, requiring about 35 minutes. Next, participants were redirected from the WB-GTH site to the NutritionQuest website where they completed the Block 2005 Food Frequency Questionnaire (FFQ), which required from 30 to 40 minutes. Following each participant’s completion of the FFQ, project staff sent the participant a digital bathroom scale and a pedometer for tracking daily steps taken for 1 week, as described below. Participants were sent 2 email reminders after each assessment component if they did not return to complete the next component within 7 days of the possible completion date.

#### Nutrition  

Web-health users completed the Block 2005 FFQ (NutritionQuest, Berkeley, CA) [36] online. FFQ estimates of intake of daily total fat, percent kcal from fat, daily total fiber, daily fiber grams from beans, daily fiber from fruits and vegetables, daily servings of fruits, daily servings of vegetables, and daily servings of fruits and vegetables combined were examined.

#### Physical Activity 

Web-health users used a pedometer (Yamax Digi-walker SW-200, San Antonio, TX) and completed a 7-day walking log provided by the project to record their daily steps taken and their daily minutes walked for exercise. They were to return to the WB-GTH website at the end of 10 days to allow for delivery time and to report at least 4 days of daily steps and minutes walked. The mean (SD) number of days at which participants returned was 15.90 days (6.98 days) excluding 6 participants who began their logs more than 60 days after the logs had been sent. The mean (SD) days of daily steps and minutes walked participants reported at this time was 6.09 days (1.20 days). Mean daily steps and mean daily minutes walked (total steps or total minutes/days recorded) were examined.

#### Social Cognitive Variables 

The Health Beliefs Survey (HBS) [19,20], administered online, measured baseline nutrition- and physical activity-related social support, self-efficacy, outcome expectations, and self-regulation (see Table 1).

**Table 1:** Health Beliefs Survey: Scale descriptions and internal consistency estimates of social cognitive measures

Variable Description and Subscale			Number of Items	Cronbach Alpha^a^
**Food beliefs survey**				
	**Social support**			
		Family	8	.89
		Friends	7	.88
	**Self-efficacy**			
		Eating healthy foods	16	.91
		Avoiding high fat and high sugar foods	6	.83
		Planning and tracking intake	10	.96
	**Positive physical outcome expectations**		7	.89
	**Negative social outcome expectations**		5	.72
	**Negative self-evaluative outcome expectations**		7	.66
	**Self-regulation**			
		Planning and tracking	11	.91
		High fat and high sugar foods	13	.90
		Healthy food choices	8	.90
**Physical activity beliefs survey**				
	**Social support**			
		Family	8	.94
		Friends	8	.96
	**Self-efficacy to face social, emotional, logistical barriers**		22	.95
	**Outcome expectations**			
		Positive physical outcome expectations	7	.89
		Positive self-evaluative outcome expectations	10	.89
		Negative social outcome expectations	6	.85
	**Self-regulation**			
		Set goals and plan physical activity	9	.91
		Track physical activity	5	.85
		Increase physical activity enjoyment	3	.77

^a^ Coefficient of internal consistency

### Statistical Analysis

Latent-variable structural equation modeling (SEM) with LISREL 8.8 (Scientific Software International, Inc, Lincolnwood, IL) [37] assessed the extent to which SCT variables contributed to the nutrition and physical activity behavior of Web users interested in participating in a Web-based nutrition, physical activity, and weight gain prevention intervention. Model fit was evaluated with the Normed Fit Index (NFI) and Nonnormed Fit Index (NNFI) > .90, root mean square error of the approximation (RSMEA) < .05 (*P* close fit > .05). Chi-square was not used in deference to the large sample size. Latent variables were measured with scores from the FFQ, HBS, and the 7-day walk log. With few exceptions, the distributions of measure scores were skewed or displayed unacceptable kurtosis; measures were normalized using the Blom proportional estimate formula in SPSS version 17.0 (SPSS Inc, Chicago, IL). Additional variables were similarly normalized to retain a consistent unit of measurement within latent variables.

## Results

### Enrolled Participants

Of 1307 Web users eligible to participate in the WB-GTH baseline assessment phase, 963 (74%) consented to become part of the study. Eligible Web users took an average of about 1 day (mean 1.38 SD 4.51) to enroll and to consent to become part of the study, but this ranged from 1 to 52 days.

Of the 1307 eligible users, 26% (344) either failed to complete consent procedures going no further in the online enrollment process (n = 297) or clicked and confirmed the box “I decline to be part of the study” that was available on all pages of the online consent form (n = 47). Participants who did not consent did not differ in age, racial/ethnic background, gender, or BMI from those who did consent to participate in the study (alpha = .05). 

Enrolled Web-health participants had a mean (SD) age of 44.40 years (11.03 years), 83% (803/963) were female, and 91% (873/963) were white. The sample was well educated: participants had completed a mean (SD) of 17.08 (3.3) years of education. Participants also had a median annual household income of about US $85,000, 83% (803/963) were overweight or obese, and 69% (507/735) of those completing the 7-day walk log had step counts in the sedentary to inactive range (ie, < 7500 steps/day). The average (SD) number of steps per day among participants was 6480.31 (2350.86). Most participants lived in the United States, but a small number (42) were Canadian residents. Although 51% (488/963) of participants lived in Virginia, the research location, most states were represented in the study (no participants lived in South Dakota, Louisiana, Rhode Island, or Iowa).

Of the 963 Web users participating, 731 completed all components of the baseline assessment in 11 to 135 days. The average (SD) number of days to completion of the baseline assessment was 22.83 (12.62) days. Although the assessment was designed to be completed across 8 days (1.25 hours online, plus the 7-day walking log), only a small percentage followed the prescribed timeline; 95% (694) completed the assessment within 45 days of enrollment. There were no demographic, social cognitive, or nutritional differences between participants with all assessment components and those without, with one exception. Participants who dropped out of the study prior to completion appeared to have slightly lower self-efficacy for making changes in their nutrition behavior that those who completed. Among those who dropped out during the assessment, the mean (SD) self-efficacy score for avoiding high fat and high sugar foods was 67.83 (22.19) versus 71.97 (19.49) among those who did not drop out (F_935,1_ = 5.06, *P* = .03) and the mean (SD) self-efficacy score for tracking nutrition was 79.61 (22.63) among those who dropped out versus 82.87 (17.04) among those who did not (F_935,1_ = 6.79, *P* = .009).

### Nutrition Characteristics of Web-Health Users

#### Fat, Fiber, Fruit, and Vegetable Consumption

Table 2 contains the means and standard deviations of Web users’ consumption of fat, fiber, and fruit and vegetable servings. Overall, Web users’ dietary consumption was higher in fat and lower in fruits, vegetables, and dietary fiber than recommended. Most, 56% (494/884), consumed more than the generally recommended 65g of total fat/day, 36% (322/884) reported consuming more than 80g of total fat/day, and almost 20% (172/884) reported consuming more than 100g of total fat/day. Only 13% (115/884) of Web-health users consumed the recommended level of 30% or fewer calories from fat; 78% (690/884) reported getting more than half their calories from fat. Similarly, 13% (115/884) of users met recommended levels of fiber intake (ie, at least 25 g/day); 68% (601/884) reported consuming fewer than 20g of fiber/day. Web-users reported somewhat better levels of fruit and vegetable consumption compared with consumption of fiber and fat with 29% (256/884) of participants consuming the recommended level of at least 5 servings/day and almost half consuming at least 4 servings but the remaining users consuming 3 or fewer servings/day.

#### Nutrition-Related Social Cognitive Characteristics

Participant means and standard deviations on the Food Beliefs Survey section of the HBS are reported in Table 2. Web-health users’ responses to the nutrition social support items suggested that they perceived their family members and friends as being fairly neutral in their support of healthier food choices (ie, scores just under 3 on the 5-point Likert-type scale). Web-health users had positive, but not complete, confidence in their ability to eat healthier foods, avoid high fat and high sugar foods, and keep track of their food choices (ie, scoring 71 to 82 on the 100-point Self-efficacy scale). They seemed to agree that their physical health (eg, weight, blood pressure, and appearance) would improve with healthier food choices (ie, scoring on average 4.3 on a 5-point Positive Physical Outcome Expectations scale). Participants were less concerned (ie, scoring on average approximately 2.9 on each 5-point scale), however, that such changes would result in negative social and self-evaluative outcomes (eg, having less time and energy for others and other activities and dissatisfaction with healthier foods).

**Table 2:** Nutrition and physical activity behavior and social cognitive characteristics of inactive but otherwise healthy adults enrolling in a Web-based health promotion intervention trial

		Mean	SD	Range
**Nutrition characteristics**				
	Total fat per day	77.79	41.96	19.20 -249.82
	Percent kcals from fat	36.51	5.92	17.13 - 60.71
	Total fiber g/day	17.74	7.35	1.11 - 44.91
	Fiber from beans g/day	2.36	2.04	0 - 18.34
	Fiber from fruits and vegetables g/day	6.95	4.12	0.10 - 29.55
	Vegetables servings/day	2.95	1.85	0.02 - 12.87
	Fruit servings/day	1.08	0.80	0.01 - 4.73
	Fruit and vegetables servings/day	4.03	2.33	0.04 – 12.47
	Family social support	2.71	0.85	1 - 5
	Friends social supports	2.85	0.79	1 - 5
	Self efficacy: eating healthy foods	76.18	17.46	9.38 - 100
	Self efficacy: tracking nutrition	82.16	18.46	0 - 100
	Self efficacy: avoid high fat and high sugar foods	71.06	20.18	11.33 - 100
	Positive physical outcome expectations	4.33	0.61	1 - 5
	Negative self-evaluative outcome expectations	2.89	0.78	1 - 4
	Negative social outcome expectations	2.93	0.84	1 - 4
	Self-regulation of eating healthy food choices	2.72	0.83	1 - 5
	Self-regulation of high fat and high sugar foods	2.97	0.81	1 - 5
	Planning and tracking nutrition choices	1.99	0.86	1 - 5
**Physical activity characteristics**				
	Steps per day	6485.78	2352.54	605.40 - 18,629.43
	Minutes walked for exercise per day	13.19	12.80	0 - 70
	Family social support	2.43	1.04	1 - 5
	Friends social support	2.80	0.96	1 - 5
	Self efficacy in face of barriers	64.61	19.57	9 - 100
	Positive self-evaluative outcome expectations	17.25	4.69	1 - 25
	Positive physical outcome expectations	20.56	4.57	1 - 25
	Negative social outcome expectations	10.51	4.93	1 - 25
	Set goals and plan physical activity	2.01	0.80	1 - 5
	Increase physical activity enjoyment	1.79	0.83	1 - 5
	Track physical activity	1.53	0.70	1 - 5

Finally, Web-health users indicated they had never-to-seldom (rated 1 and 2, respectively, on the Self-regulation scale) planned or tracked healthier food choices in the 3 months before the assessment (eg, keep track of high fat snacks or plan to eat fruit for breakfast). They reported that they occasionally (rated 3 on the scale) did things to reduce fat and sugar and increase healthier food choices (eg, drink water instead of sodas or eat fruit for dessert).

### Physical Activity Characteristics of Web-Health Users

#### Daily Step Counts and Minutes Walked

The Web users in the study were selected based on self-reports of exercising less than 20 minutes 3 times a week in the month preceding the assessment.

##### Daily Steps

Among the inactive participants, average steps logged over 7 days fell within the low active range [38] (see Table 2); 27% (198/735) of the Web-health users took fewer than 5000 steps/day, 42% (309/735) took 5000 to 7499 steps/day, 24% (228/735) took 7500 to10,000 steps/day, and 8% (56/735) took more than 10,000 steps/day.

##### Daily Minutes Walked for Exercise

Web-health users logged an average of less than a quarter of an hour in daily walking (see Table 2); 41% (299/735) logged virtually no walking (< 3 minutes/day). On the other hand, 22% of the sample logged 20 minutes or more/day in walking (169/735).

#### Physical Activity-Related Social Cognitive Characteristics

Participants’ means and standard deviations from the Physical Activity Beliefs Survey portion of HBS can be found in Table 2. Web users interested in a program to help them become more active generally did not perceive their friends and family members as taking steps to being physically active themselves (ie, social support scores of < 3.0 on the 5-point scale). Physical-activity self-efficacy scores indicated that Web-health users had some confidence in their ability to increase physical activity in the face of social, emotional, and logistical barriers (ie, the mean score was about 65 on a 100-point scale). Within the self-efficacy items on the Physical Activity Beliefs Survey, however, participants’ responses varied. Compared to Web-users’ higher mean (SD) score of 80.02 (19.75) on items regarding managing a walking routine (ie, keeping track of walking, making plans to exercise, and resuming walking after a break), their mean (SD) score of 54.75 (23.52) indicated they were less confident in their abilities to deal with the social aspects of becoming more active (ie, finding someone to walk with, exercising when family wanted more time, or socializing only after meeting exercise goals) (*t*_936_ = –40.38, *P* *<* .001).

Web-health users expected that increasing physical activity would result in health benefits (ie, their mean score was 21 on a 25-point Positive Physical Outcome Expectations scale) and would be good for their mental and physical state (ie, their mean score was 17 on a 25-point Positive Self-evaluative Outcome Expectations scale). Participants were more neutral in their expectations that being more active would interfere with the time they would have for others and other activities (ie, a mean score of 10 on the 25-point Negative Social Outcome Expectations scales).

Overall, Web-health users indicated they had never or seldom (rated 1 and 2 on the scale, respectively) implemented physical activity self-regulation strategies in the 3 months before the assessment (see Table 2). The Web-health users did not track their physical activity (ie, frequency, duration, or intensity of exercise) but were more likely to set goals and plan for being physically active (*t*_936_ = 26.66, *P* *<* .001)

### Social Cognitive Determinants of Web Users’ Nutrition and Physical Activity Levels

#### Nutrition Models

**Figure 3:**
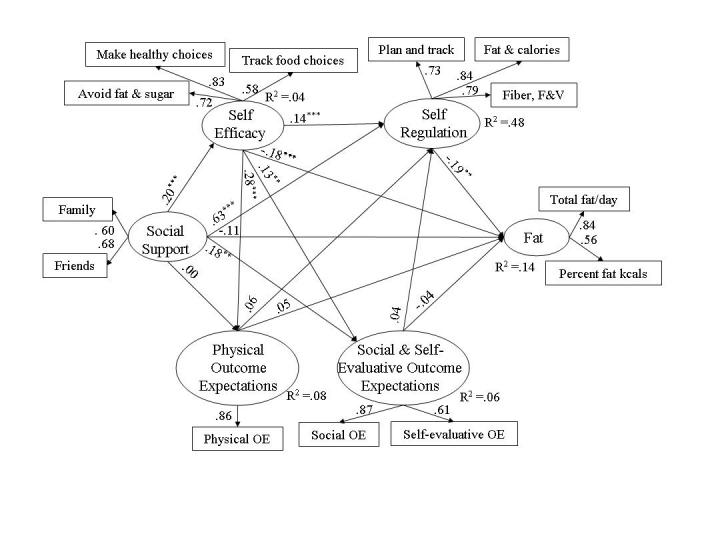
Social cognitive model of fat consumption among Web-health users where * signifies *P* < .05, ** signifies *P* < .01, and *** signifies *P* < .001

**Figure 4:**
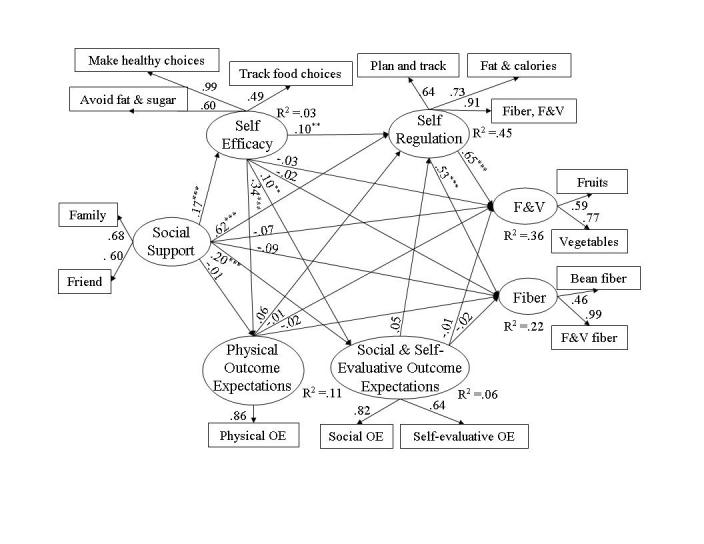
Social cognitive model of fiber, fruit, and vegetable consumption among Web-health users where * signifies *P* < .05, ** signifies *P* < .01, and *** signifies *P* < .001

Structural equation analyses evaluated behavioral and social cognitive variables simultaneously to determine how well the SCT models of fat (see Figure 3) and of fiber, fruits, and vegetables (see Figure 4) fit the data collected from the Web-health users. Fit was good for each model; specifically, for the fat model, RMSEA = .045 (95% confidence interval [CI] .04 - .05), *P* (close fit) = .80, NFI = .97, and NNFI = .97. For the fiber, fruit and vegetables model fit indicators were RMSEA = .048 (95% CI .04 - .06), *P* (close fit) = .66, NFI = .97, and NNFI = .96. The SCT models differed in the amount of variance each explained, which was 14% of fat intake, 22% of fiber intake, and 36% of fruits and vegetables intake. The completely standardized parameter coefficients associated with direct effects of the latent variables in the models are illustrated in Figures 3 and 4. A variable’s direct effect is the portion of its total effect that is independent of other variables in the model; a variable’s indirect effect is the portion of its total effect that is dependent on other variables (covariance matrices and factor loadings associated with the analyses are available from author EA). 

##### Social Support and Dietary Intake

Social support from friends and family made a strong contribution (ie, beta _total_ > .20 [39]) to healthier nutrition: Web users who perceived that important others were attempting healthier eating had lower levels of fat (beta _total_ = -.28, *P* < .001) and higher levels of fiber (beta _total_ = .25, *P* *<* .001) and fruits and vegetables (beta _total_ = .34, *P* *<* .001). The total effect of social support on Web-health users’ fat intake was largely indirect (beta _indirect_ = -.17, *P* < .001, indirect/total ratio = .68) through social support’s effect on other model variables influencing fat levels (ie, self-efficacy, beta _total_ = .20, *P* *<* *.*001 and self-regulation, beta _total_ = .67, *P* < .001). On the other hand, the effect of social support on fiber and fruits and vegetables was entirely indirect (fiber, beta _indirect_ = .34, *P* *<* .001, indirect/total ratio = 1.36 and fruits and vegetables, beta _indirect_ = .42, *P* *<* *.001*, indirect/total ratio = 1.23) through self-efficacy (beta _total_ = .17, *P* < .001) and self-regulation (beta _total_ = .65, *P* < .001). The large positive indirect effects of social support counteracted small, insignificant negative direct effects on fiber, fruit, and vegetable consumption (see Figure 4).

##### Self-efficacy and Dietary Intake

Fat intake was also strongly associated with self-efficacy; Web-health users with higher confidence in their ability to make healthier food choices, plan and track food intake, and avoid high fat and high sugar foods reported lower levels of fat on the FFQ (beta _total_ = -.21, *P* *<* .001). Self-efficacy did not influence Web users intake of fiber (beta _total_ = .05, *P* = .27) and fruits and vegetables (beta _total_ = .05, *P* = .23). Although self-efficacy influenced outcome expectations (negative outcome expectations, beta _total_ = .13, *P* = .006; positive outcome expectations, beta _total_ = .28, *P* *<* .001) and self-regulation (beta _total_ = .16, *P* *<* .001) in the fat model, the effect of self-efficacy on fat intake was largely direct (ie, beta _indirect_ = -.02, *P* = .25; indirect/total ratio = .10).

##### Outcome Expectations and Dietary Intake

Negative and positive outcome expectations did not exert total effects on the content of Web users’ food intake. This was true for fat (negative outcome expectations, beta _total_ = -.04, *P* = .37; positive outcome expectations, beta _total_ = .03, *P* = .47), fiber (negative outcome expectations, beta _total_ = .01, *P* = .87; positive outcome expectations, beta _total_ = .02, *P* = .59) and fruits and vegetable (negative outcome expectations, beta _total_ = .02, *P* = .66; positive outcome expectations, beta _total_ = .03, *P* = .60). Outcome expectations also did not influence self-regulation as hypothesized by the SCT model (see Figures 3 and 4).

##### Self-regulation and Dietary Intake

Enactment of self-regulatory behaviors was a moderate (ie, beta = .10 - .19) predictor of Web-health users’ fat intake and a strong predictor of fiber, fruits, and vegetable consumption. Planning and tracking and using strategies to increase healthy food choices and to avoid high fat and sugar foods led to lower levels of fat (beta _total_ = -.19, *P* = .008), higher levels of fiber (beta _total_ = .53, *P* < .001), and higher levels of fruits and vegetables (beta _total_ = .65, *P* < .001) in Web-health users’ food intake. 

#### Physical Activity Model

**Figure 5:**
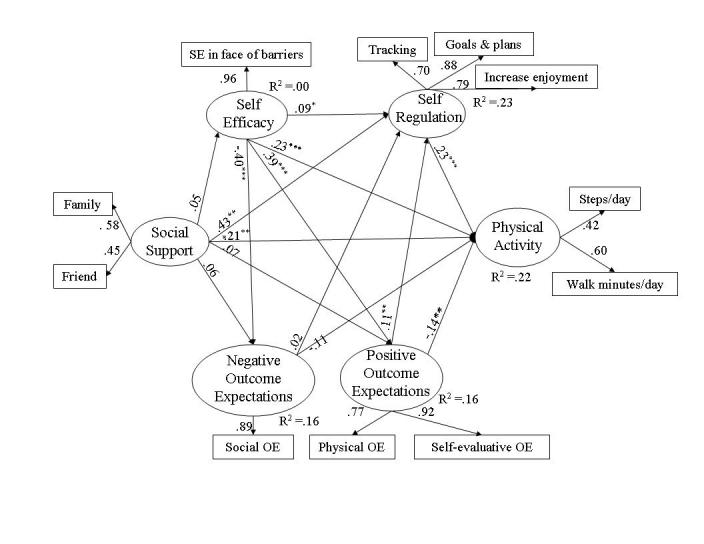
Social cognitive model of physical activity among Web-health users where * signifies *P* < .05, ** signifies *P* < .01, and *** signifies *P* < .001

Structural equation analyses indicated good fit of the SCT model to physical activity data from Web-health users with fit indicators of RMSEA = .029 (95% CI .01 - .04), *P* (close fit) = .99, NFI = .98, and NNFI = .99. The SCT model explained 22% of the variance observed in physical activity levels. The completely standardized parameter coefficients associated with direct effects of the latent variables in the models are displayed in Figure 5.

##### Social Support and Physical Activity

Social support from friends and family contributed substantially to Web-health users’ physical activity levels (beta _total_ = .30, *P* < .001), an effect that was partly indirect through self-regulation (beta _indirect_ = .10, *P < .001*, indirect/total ratio = .33). Although social support did not influence self-efficacy (beta _total_ = .05, *P* = .37) or outcome expectations (negative outcome expectations, beta _total_ = .04, *P* = .48; positive outcome expectations, beta _total_ = .09, *P* = .10), social support was strongly predictive of whether Web users engaged in self-regulatory behavior (beta _total_ = .45, *P* < .001). Participants who perceived support from their friends and families for physical activity were more likely to set goals, plan, and self-monitor their own activity levels. The effect of social support on self-regulation was largely direct (beta _indirect_ = .02, *P* = .10, indirect/total ratio = .04)

##### Self-efficacy and Physical Activity

Web-health users with greater confidence in their abilities to manage the social, emotional, and logistical barriers to walking on a regular basis were more active; this strong effect was almost entirely direct (beta _total_ = .25, *P* < .001; beta _indirect_ = .02, *P* = .49; indirect/total ratio = .08). In addition to physical activity, self-efficacy moderately influenced self-regulation (beta _total_ = .13, *P* < .001) and was a strong predictor of outcome expectations in the model (negative outcome expectations, beta _total_ = -.40, *P* *<* .001; positive outcome expectations, beta _total_ = .39, *P* < .001). Participants with confidence in their abilities to maintain an active lifestyle were more likely to expect to reap the benefits from becoming more active and were more likely to engage in self-regulatory behavior.

##### Outcome Expectations and Physical Activity

As in the nutrition models, outcome expectations did not exert total effects on Web users’ physical activity (negative outcome expectations, beta _total_ = -.11, *P* = .09; positive outcome expectations, beta _total_ = -.11, *P* = .10). Positive outcome expectations (physical and self-evaluative), however, did have a significant but negative direct effect on physical activity (beta _direct_ = -.14, *P* = .02, which was somewhat counterbalanced by a small, positive indirect effect (beta _indirect_ = .03, *P* = .06) through positive outcome expectations' effects on self-regulation (beta _total_ = .11, *P* = .007).

##### Self-regulation and Physical Activity

Enactment of self-regulatory behaviors was a strong predictor of Web-health users’ physical activity. Setting activity goals and making plans, adjusting routines to make activity more enjoyable, and tracking daily activity led to higher levels of walking (beta _total_ = .23, *P* = .003).

## Discussion

Web-health users visiting and ultimately enrolling in an entirely online nutrition, physical activity, and weight gain prevention intervention study (WB-GTH) were generally middle-aged, well-educated, upper middle class women whose poor diet and exercise habits put them at risk of obesity, heart disease, some cancers, and diabetes. Nutrition and physical activity behavior among the Web users when they enrolled was predicted by the support they perceived from others for healthier behavior, the extent to which they used self-regulatory strategies essential to maintaining a healthy lifestyle, and, to a certain extent, their self-efficacy for making healthier choices.

Designed for inactive but otherwise healthy Web users, the WB-GTH website attracted almost 4700 participants over 12 months of recruitment. Participants were directed to the site by advertisements through print and online media and online social and professional networks. A high percentage of those visiting the site (3944 or 84%) registered to see if they were eligible for the study. As observed in a national sample of Web-health users [1], registered WB-GTH users were largely middle-aged, non-Hispanic white, and female. For the parent study, elderly, unhealthy, and morbidly obese adults were excluded from the sample; it appears exclusionary criteria may have disproportionately eliminated non-white participants, perhaps reflecting higher rates of obesity and disease in the African American population [40]. Thus the long-term commitment, evaluation components of the research, and the eligibility criteria required for enrollment in the study limit the external validity of these findings.

The number of eligible registrants remaining in the sample shrank at each step of the enrollment and assessment process consistent with patterns described in earlier studies [4,6]. Among 1307 registrants who met eligibility requirements, about one-quarter (344) declined to participate in the study. Although only minimal information was collected from participants prior to consent, those who did not consent did not differ in age, racial/ethnic background, gender, or BMI from those who did consent to participate in the study. Consistent with the pool of registered Web users, most of the 963 users consenting to participate in the WB-GTH trial and most of the 731 users who completed all assessment components were female and non-Hispanic white. They were also well educated with at least some college education and were upper-middle class with a median annual household income of about US $85,000, consistent with other Web-based nutrition trials [41]. Reflecting the study’s inclusion criteria, the resulting sample was overweight or obese with step counts generally in the sedentary to inactive range (ie, < 7500 steps/day). Further, the vast majority did not meet guidelines for intake of fat, fiber, and fruit and vegetables.

In light of their detrimental nutrition and physical activity behaviors, Web-health users exhibited comparatively high levels of self-efficacy for making changes and of expectations that changes would have health benefits. The juxtaposition of high efficacy and expectations with low levels of healthy behavior is common. Bandura [4] suggests that self-efficacy for behavior change can be unrealistically high among individuals who lack experience in the desired, healthier behavior. Similarly, Polivy and Herman [42] have posited a false hope syndrome, which might suggest that recruits for a health-promotion intervention may be unrealistic about the benefits of behavior change (as suggested by the inverse direct relation of high positive expectations and physical activity here). Web users’ lower confidence in managing the social aspects of becoming more active, their lower levels of perceived social support for behavior change, more neutral social outcome expectations, and virtual lack of self-regulatory behaviors related to making healthy changes are more consistent with the inactivity and unhealthful diets observed in the sample. This suggests that for Web-health users who may typically have low levels of health-promoting behaviors, SCT-based interventions may temper users’ pre-intervention self-efficacy levels. 

The SCT-based structural equation models testing the relations among SCT variables and behavior provided good fit to the Web-health users’ nutrition and physical activity data (RMSEA < .05). Consistent with other research, perceived social support and engaging in self-regulatory behaviors exerted strong influences on physical activity and nutrition behavior [20,22]. Higher levels of self-efficacy also contributed to physical activity and lower dietary fat but not to higher levels of fiber, fruits, and vegetables among Web-health users. Outcome expectations did not exert a total effect on users’ nutrition behavior or physical activity. SCT interventions, then, may be more successful to the extent they help Web-health users garner support for making changes from significant others. Improved social support and subsequent increases in self-efficacy could lead directly to improvements in physical activity and nutrition behavior but would also be effective pathways for increasing the use of self-regulatory strategies essential to healthy levels of activity and food choices. Among Web-health users, even small increases in self-regulatory behaviors could be expected to have substantial impact on dietary and physical activity behaviors. Providing a platform for setting behavioral goals, planning, tracking, and providing feedback would be a considerable strength of automated, self-administered Internet-based health promotion programs.
